# Multisystem *Lomentospora prolificans* progression on fosmanogepix despite *in vitro* activity: a call for expanding clinical research on novel antifungals

**DOI:** 10.1128/asmcr.00189-25

**Published:** 2026-01-29

**Authors:** Ryan A. Cooper, Sana Arif, Arthur W. Baker, Nathan P. Wiederhold, Stefanie Sarantopoulos, Mustafa Iftikhar, Kelly Stanly, Brennan Collis, Bin Ni, Beatrice Z. Sim, John R. Perfect, Barbara D. Alexander, Madeleine R. Heldman

**Affiliations:** 1 Department of Medicine, Duke University School of Medicine 12277 , Durham, North Carolina, USA; 2 Division of Infectious Diseases, Department of Medicine, Duke University School of Medicine 12277 , Durham, North Carolina, USA; 3 Fungus Testing Laboratory, University of Texas Health Science Center 14742, San Antonio, Texas, USA; 4 Division of Hematologic Malignancies and Cellular Therapies, Department of Medicine, Duke University School of Medicine 12277 , Durham, North Carolina, USA; 5 Duke Cancer Institute, Duke School of Medicine 12277 , Durham, North Carolina, USA; 6 Department of Ophthalmology, Duke University School of Medicine 12277 , Durham, North Carolina, USA; Vanderbilt University Medical Center, Nashville, Tennessee, USA

**Keywords:** invasive fungal infection, *Lomentospora prolificans*, fosmanogepix, olorofim, publication bias

## Abstract

**Background:**

*Lomentospora prolificans* is a rare and often fatal cause of invasive mold disease (IMD), particularly in immunocompromised individuals. Treatment remains difficult due to intrinsic resistance to most antifungals and the challenges of achieving therapeutic drug levels in the central nervous system. Fosmanogepix and olorofim are novel antifungal agents with promising activity against difficult-to-treat molds, including *L. prolificans*, and show potential for excellent central nervous system penetration.

**Case Summary:**

We report a case of disseminated *L. prolificans* infection with pulmonary, ocular, and central nervous system involvement in a 73-year-old man following haploidentical hematopoietic cell transplantation for peripheral T-cell lymphoma. Despite early neutrophil engraftment, the absence of graft-versus-host disease, and treatment with fosmanogepix, his infection progressed to fatal fungal meningitis.

**Conclusion:**

This case underscores the limitations of relying on *in vitro* susceptibility results to predict clinical response in the absence of clinical breakpoints, particularly in deep-seated infections where drug penetration may be limited. While prior case reports have described successful outcomes with novel agents, publication bias may overrepresent favorable results. This case supports the urgent need for rigorous evaluation of emerging antifungal therapies in real-world settings.

## INTRODUCTION

*Lomentospora prolificans* is a rare but often fatal cause of invasive mold disease (IMD) in immunocompromised patients (1–3). The treatment of *L. prolificans* infections is challenging since isolates often demonstrate poor *in vitro* susceptibility to clinically accessible antifungals. *L. prolificans* infections frequently involve the central nervous system (CNS), making drug delivery across the blood-brain barrier an additional challenge (3, 4).

Fosmanogepix and olorofim are two first-in-class novel antifungal drugs that have demonstrated promising safety profiles and efficacy in preclinical and early-stage clinical trials for treatment of IMDs, including IMD with CNS involvement (5). Fosmanogepix is a prodrug of manogepix which targets Gwt1, an enzyme essential for trafficking of mannoproteins to the cell membrane and cell wall (5, 6). Olorofim inhibits fungal dihydroorotate dehydrogenase in pyrimidine synthesis (7, 8). Preclinical models of fosmanogepix in non-neutropenic rabbits suggest high penetration of manogepix into the CNS (9). Several case reports describe successful treatment of ocular and CNS IMDs using these novel antifungals (10, 11). While these reports are encouraging, few cases have been described in detail, and case reports are prone to publication bias in favor of positive outcomes (12, 13). We present a case of disseminated *L. prolificans* with ocular and CNS involvement in an allogeneic hematopoietic cell transplant (HCT) recipient who died of progressive infection after treatment with fosmanogepix, despite a favorable manogepix *in vitro* susceptibility result.

## CASE PRESENTATION

**Fig 1:**
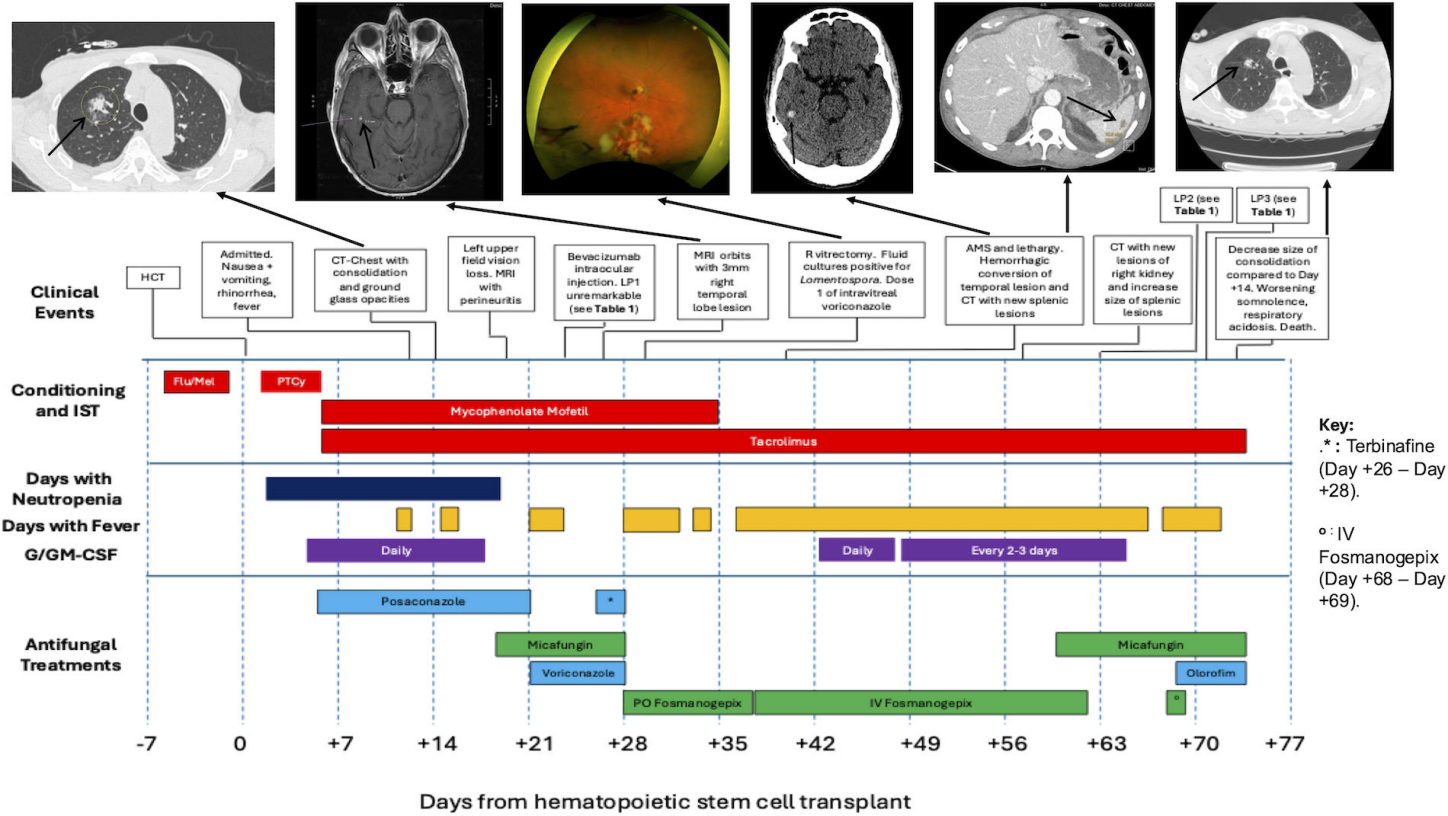
Timeline of events and antifungal treatments in the management of multisystem *Lomentospora prolificans* in a hematopoietic cell transplant recipient. Associated radiographic and ophthalmologic studies are shown above, connected to the respective dates of study. Days with fever were defined as a temperature above 100.4°F. Neutropenia was defined as absolute neutrophil count less than 0.5 × 10^9^/L. Fludarabine was administered on days −5 to −2, and melphalan was administered on day −2. Cyclophosphamide was administered on days +3 and +4. AMS, altered mental status; CT, computed tomography; Flu, fludarabine; G/GM-CSF, granulocyte/granulocyte-macrophage colony-stimulating factor; HCT, hematopoietic cell transplant; IST, immunosuppressive therapy; LP, lumbar puncture; Mel, melphalan; MRI, magnetic resonance imaging; PTCy, post-transplant cyclophosphamide.

A 73-year-old man with peripheral T-cell lymphoma underwent a haploidentical HCT with intravenous fludarabine and intravenous melphalan for conditioning. Prior to treatment, he had a normal neutrophil count without any history of severe systemic infection. He received post-transplant intravenous cyclophosphamide on days +3 and +4. Intravenous tacrolimus and oral mycophenolate mofetil were started on day +5 for graft-versus-host disease (GVHD) prophylaxis. He began antifungal prophylaxis with delayed-release oral posaconazole tablets 300 mg daily on day +5. On day +12, he developed febrile neutropenia, nausea, vomiting, rhinorrhea, and progressive cough. His serum posaconazole concentration was 0.5 mcg/L, below the targeted prophylactic concentration of ≥0.7 mcg/L (14). The posaconazole dose was increased from 300 mg daily to 400 mg daily. On day +14, computed tomography (CT) of the chest revealed a focal consolidation with surrounding ground-glass opacities and multiple additional sub-centimeter nodules (Fig. 1), prompting bronchoscopy with bronchoalveolar lavage (BAL) on day +17. Neutrophil engraftment occurred on day +18. On day +19, he woke up with new left upper visual field loss. An ocular exam revealed neovascularization of the iris, intraretinal and vitreous hemorrhages, and choroidal thickening on ultrasound in the left eye, raising concern for endophthalmitis and prompting treatment with intravitreal voriconazole 0.1 mg. Intravenous micafungin 150 mg daily was added to his treatment regimen.

On day +21, *L. prolificans* was detected in BAL cultures, prompting transition from systemic posaconazole to intravenous (IV) voriconazole (two 6 mg/kg loading doses given 12 h apart followed by 4 mg/kg every 12 h thereafter). On day +24, worsening left ocular scotoma prompted a lumbar puncture (LP). Cerebrospinal fluid (CSF) chemistries, cell counts, and microbiology tests were unremarkable (Table 1). On day +26, oral terbinafine 250 mg daily was added. Magnetic resonance imaging of the orbits showed a 3 mm enhancing lesion in the right temporal lobe (Fig. 1).

**TABLE 1:** Opening pressure and cerebrospinal fluid results in a hematopoietic cell transplant recipient with multisystem *Lomentospora prolificans* infection

	Day relative to transplant		
	Day +24	Day +63	Day +71
Opening pressure, cm H_2_O	–^*a*^	19	16
Red blood cell count, cells/µL (reference < 0/µL)	558	0	80
Nucleated cell count, cells/µL (reference 0–5/µL)	0	648	500
% Neutrophils (reference 0–6%)	Not applicable	82	70
% Lymphocytes (reference 40–80%)	Not applicable	13	25
% Monocytes	Not applicable	4	5
Protein (mg/dL) (reference range 15–50 mg/dL)	54	227	353
Glucose (mg/dL)^*b*^	73	43	45
(1-3)-Beta-D-glucan (pg/mL)	–	>500	–
Multiplex polymerase chain reaction (PCR)^*c*^	No targets detected	HHV-6 detected^*d*^	–
Human herpesvirus-6 (HHV-6) PCR	–	Not detected	–
Herpes simplex virus −1 (HSV-1) and −2 (HSV-2) PCR	–	Not detected	–
Cryptococcal antigen	Not detected	Not detected	Not detected
Aerobic culture	No growth at 5 days	No growth at 5 days	No growth at 5 days
Fungal culture	No growth at 21 days	No growth at 21 days	No growth at 21 days
Mycobacterial culture	–	No growth at 42 days	–

^*a*^ – indicates that the test was not performed.

^*b*^ Concurrent plasma glucose testing was not performed.

^*c*^ Multiplex PCR included targets for *Cryptococcus neoformans*, Cytomegalovirus, Enterovirus, *Escherichia coli*, *Haemophilus influenzae*, Herpes simplex virus 1, Herpes simplex virus 2, Human herpesvirus 6, Human parechovirus, *Listeria monocytogenes*, *Neisseria meningitidis*, *Streptococcus agalactiae*, *Streptococcus pneumoniae*, Varicella-zoster virus.

^*d*^ HHV-6 was detected on CSF multiplex PCR panel, but HHV-6 was not detected on quantitative HHV-6-specific PCR performed on the same specimen. A same-day quantitative plasma HHV-6 PCR was negative. Together, these factors argue against HHV-6 encephalitis (15).

Due to concerns about multifocal *L. prolificans* infection (lung, eyes, and brain), the historically poor prognosis of disseminated *L. prolificans* infections, and reports of favorable clinical outcomes with fosmanogepix for difficult-to-treat IMDs (10, 11, 16, 17), fosmanogepix was obtained through an expanded access program (EAP). On day +28, oral fosmanogepix 800 mg daily was started, and voriconazole, micafungin, and terbinafine were discontinued. Combination antifungal treatment with fosmanogepix plus liposomal amphotericin B was deferred because *Lomentospora* spp. are intrinsically resistant to amphotericin, and liposomal amphotericin B carries a significant risk of nephrotoxicity (18). Combination therapy with fosmanogepix plus either a triazole or terbinafine was also deferred because *Lomentospora* spp. often demonstrate poor *in vitro* susceptibility to triazoles and terbinafine, and because there are no clinical data to support the use of fosmanogepix in combination with these agents (19). Day +28 ophthalmological exam revealed interval development of intraretinal hemorrhages and peripheral vascular sheathing in the right eye. Given progression to bilateral intraocular involvement, he underwent right vitrectomy with intravitreal voriconazole injection (0.1 mg) on day +30 (Fig. 1). Vitreous fluid culture ultimately revealed *L. prolificans,* and intravitreal voriconazole injections (0.1 mg/injection) were continued two to three times weekly.

On day +36, he developed delirium with decreased interpersonal interactions. CT of the brain showed hemorrhagic conversion of the right temporal lesion (Fig. 1). Due to ongoing delirium and intermittent diarrhea and emesis, oral fosmanogepix was transitioned to the IV formulation at 600 mg daily. Abdominal and chest CT on day +39 showed scattered hypoattenuating splenic lesions suspicious for metastatic fungal infection (Fig. 1), along with mild interval improvement in bilateral chest nodules.

On day +43, antifungal susceptibilities for the BAL isolate of *L. prolificans* were determined using the Clinical and Laboratory Standards Institute (CLSI) M38 standard broth dilution method (20). The manogepix minimum effective concentration (MEC) was ≤0.008 mcg/mL, the olorofim minimum inhibitory concentration (MIC) was 0.06 mcg/mL, and all -azole MICs were >16 mcg/mL (Table 2). Granulocyte-macrophage colony-stimulating factor was added on day +43 and administered every 1–3 days until day +65.

**TABLE 2:** Antifungal minimum inhibitory concentrations (MIC) for azoles, terbinafine, and olorofim and minimum effective concentration (MEC) for manogepix against *Lomentopora prolificans* isolated from bronchoalveolar lavage fluid culture^a^

Drug	MIC/MEC (mcg/mL)
Posaconazole	>16
Voriconazole	>16
Isavuconazole	>16
Terbinafine	>2
Manogepix	≤0.008
Olorofim	0.06

^a^ Testing was performed by the Clinical Laboratory Standards Institute (CLSI) M38 broth microdilution method (20). MICs were read as the lowest concentrations resulting in 100% inhibition of growth, and the MEC was read as the lowest concentration of manogepix that resulted in morphologic changes (i.e., short, stubby, abnormally branched hyphae) (5).

By day +55, his mental status continued to fluctuate. The patient was minimally conversant and intermittently able to follow commands. Fosmanogepix-mediated neurotoxicity was considered a potential cause for his deterioration, and fosmanogepix was held from day +62 to day +68 (Fig. 1). LP was performed on day +63 and revealed a new CSF pleocytosis (648 nucleated cells/µL), hyperproteinorrachia (CSF protein 227 mg/dL), and elevation of CSF (1-3)-Beta-D-glucan (>500 pg/mL), findings consistent with fungal meningitis (Table 1).

Due to clinical worsening and ongoing fevers, fosmanogepix was stopped, and olorofim 150 mg twice daily was started on day +70, when it became accessible through an EAP. Olorofim tablets were dissolved in warm water and administered via enteric tube. Intravenous micafungin 100 mg daily was administered with olorofim for yeast prophylaxis. Repeat CSF studies on day +71 were relatively unchanged from those on day +62 (Table 2). The patient developed respiratory acidosis, transitioned to comfort-focused care, and died on day +73.

## DISCUSSION

Our patient’s ocular, CNS, and pulmonary *L. prolificans* infection likely progressed to fatal fungal meningitis. Progression occurred despite a manogepix *in vitro* susceptibility result that appeared to be more favorable than those for voriconazole and terbinafine, a guideline-supported combination antifungal regimen (1, 21, 22). Olorofim was not used until his overall condition had deteriorated, limiting interpretation of the drug’s effectiveness.

Several factors may have contributed to the progression of infection on fosmanogepix. First, the inherent immune deficiencies related to allogeneic HCT and the virulent nature of *Lomentospora* may have portended a poor outcome, regardless of antifungal treatment (1–3, 23). However, this patient’s post-transplant course was otherwise uncomplicated, with timely and sustained neutrophil engraftment, no GVHD or corticosteroid use, and no other significant medical comorbidities associated with poor IMD outcomes such as diabetes, chronic lung disease, cirrhosis, or chronic kidney disease (23). Second, the availability of fosmanogepix in the ocular and meningeal spaces may not have been optimal. While preclinical data of fosmanogepix in non-neutropenic rabbits demonstrated significant CNS penetration, the pharmacokinetics of manogepix in humans have not been well studied outside of plasma (9, 24). Our patient started oral fosmanogepix at 800 mg daily without preceding intravenous loading doses, which may have delayed reaching a manogepix steady state (25). It is possible that suboptimal absorption of oral fosmanogepix during the first eight days of treatment could have reduced manogepix tissue exposure, although disease progressed despite transitioning to the intravenous formulation for an additional 26 days of therapy. Third, our patient was treated with fosmanogepix monotherapy without a concomitant antifungal for most of his treatment course. Preclinical models indicate that combination therapy of fosmanogepix with liposomal amphotericin B may be more effective than either agent alone, suggesting a role for synergy (5, 26). However, *in vitro* testing for synergistic antifungal combinations has not been standardized, and such testing may not predict antifungal combinations that work synergistically in the clinical setting (27, 28). Finally, even when obtained by standardized methods, low *in vitro* MIC/MECs do not always predict clinical response to antifungals, and the CLSI now considers all *Lomentospora prolificans* strains to be intrinsically resistant to amphotericin B (18). Counterproductive host inflammatory responses may negatively affect clinical outcomes during antifungal treatment despite high *in vivo* activity, and source control may facilitate clinical improvement independent of antifungal therapy (29, 30). Unfortunately, because of the diffuse nature of our patient’s disseminated infection, surgical methods to achieve source control were not feasible.

The fosmanogepix endpoint for susceptibility testing was the MEC, which measures the drug’s ability to induce morphological changes rather than inhibit growth (31). Therefore, fosmanogepix might be considered more fungistatic than fungicidal, with limited capacity to eradicate our patient’s fungal infection, which had already disseminated to the CNS before fosmanogepix was initiated. However, fosmanogepix reduced fungal burden, as measured by conidial equivalents, and improved survival in mice with disseminated *Scedosporium* and *Fusarium* harboring manogepix MECs higher than that of our patient’s isolate (32). Thus, a low MEC measurement does not necessarily predict fungicidal activity, but a low MEC to fosmanogepix may still translate into a reduction in conidial burden for certain molds.

EAPs are an important mechanism for accessing novel antifungals. Unlike clinical trials, however, EAPs lack a formal process for defining treatment success compared with standards of care. In a recent phase 2, open-label clinical trial, fosmanogepix had an acceptable safety profile among the 21 participants with proven/probable IMD. Mortality at 42 days was 25%, comparable to mortality estimates in patients treated with amphotericin B or mold-active azoles in historical clinical trials (24). Only one participant had *L. prolificans*, and few details of the case were reported. In many clinical trials for antifungals in immunocompromised populations, small study size, heterogeneity of underlying comorbidities, and diversity of fungal pathogens complicate the translation of results into clinical practice.

Challenges in antifungal clinical trial design, implementation, and accessibility encourage clinicians to rely on anecdotal experience and case reports to guide their practice. Case reports are prone to publication bias, favoring cases with positive clinical outcomes (12, 13). An early case report of fosmanogepix use described resolution of brain lesions attributed to disseminated *Fusarium lactis* in a patient with acute leukemia (10). Another patient with GVHD and *Aspergillus calidoustus* endocarditis, complicated by cerebral emboli, survived this serious infection with resolution of a persistently positive serum galactomannan following treatment with fosmanogepix (11). In both case reports, the apparent treatment success may have been influenced by other clinical factors (i.e., neutrophil recovery and mitral valve replacement, respectively), clouding the relationship between antifungal effect and clinical outcomes. Nonetheless, promising anecdotes encouraged us to pursue fosmanogepix for our patient and also influenced recommendations to include fosmanogepix in combination with liposomal amphotericin B and voriconazole for triple therapy targeting *Fusarium solani* meningitis during an outbreak (17).

This case is one of the earliest descriptions of a severe *L. prolificans* infection treated with novel antifungals. This single report of a negative outcome should not deter further study and development of novel antifungals. Instead, we share this experience to balance potential publication biases and to emphasize the potential limitations of antifungal *in vitro* susceptibilities for predicting clinical outcomes. At the time of this publication, clinical trials investigating fosmanogepix are ongoing, one of which includes a salvage treatment arm that is open to patients with invasive *L. prolificans* infections (33). Continued expansion of robust clinical trial platforms is necessary to provide patients with early access to novel therapeutics and to systematically evaluate clinical efficacy.
